# The complete chloroplast genome sequence of *Oryza rhizomatis* (Poaceae)

**DOI:** 10.1080/23802359.2017.1357453

**Published:** 2017-07-29

**Authors:** Fang Liu, Yan Zhao, Dengjie Luo, Dengwei Hong, Rongbai Li

**Affiliations:** State Key Laboratory for Conservation and Utilization of Subtropical Agro-bioresources, Agricultural College Guangxi University, Nanning, China

**Keywords:** *Oryza rhizomatis*, the genus *Oryza*, chloroplast genome

## Abstract

The complete chloroplast genome sequence of *Oryza rhizomatis* (GenBank accession number: KX085497) was generated by de novo assembly with low-coverage whole-genome sequence data. The genome was 134,796 bp in length, containing a pair of inverted repeat (IRa and IRb) regions of 20,818 bp, which were separated by a large single-copy (LSC) region of 80,829 bp and a small single-copy (SSC) region of 12,330 bp, respectively. The genic regions account for 43.77% of whole cpDNA, and the AT content of the cpDNA was 60.99%. The *O. rhizomatis* cpDNA encodes 112 unigenes (79 protein-coding genes, four rRNA genes, and 29 tRNA genes). Eighteen genes contain introns, *ycf3* contains two introns, and the rest of the gene contains one intron; *rps12* is trans-spliced, one of its exons is in the LSC region (5′_end) and the two reside in the IR regions (3′_end) separated. A pair of gene *ndhH,* due to the 5′ part of *ndhH* which overlaps the IR/SSC junctions, was two unique genes. The four rRNA genes are all located in the IR. Phylogenomic analysis showed that *O. rhizomatis* is closely related to *O. officinalis*. The new data will help to determine the phylogenetic placement of the genus *Oryza* and fill gaps in our understanding of *Oryzae* biology.

Rice is the most important crop in the world, as a major source of nutrition for more than half the global population, cultivated rice is one of the world's most important cereal crops (Vaughan et al. 2003). The *Oryza* genus has two cultivated species and about 21 wild relatives and based on chromosome paring, these *Oryza* species are divided into 10 genome types namely AA, BB, CC, BBCC, EE, FF, GG, CCDD, HHJJ, and HHKK (Ge et al. 1999).

At present, some complete cp genomes belonging to Rice genus have been available in NCBI GenBank (http://www.ncbi.nlm.nih.gov/genbank), while the *Oryza rhizomatis* cp genome has not been reported. In this study, we determined the complete chloroplast DNA sequence of *Oryza rhizomatis* **(**IRGC Acc. No. 103417), a wild rice, by using next-generation sequencing technology. We filtered and assembled the complete cp genome with CLC Genomics Workbench v3.6 software (CLC Genomics Workbench v3.6 2010). The complete cp genome sequence with gene annotations was submitted to the GenBank (GenBank accession number: KX085497). The genome consists of 134,796 bp, is a typical circular double-stranded DNA molecule with a quadripartite structure common to most land plant genomes with large single-copy (LSC) regions (80,829 bp) and small single copy (SSC) regions (12,330 bp) separated by two IR copies (20,818 bp).

The *O. rhizomatis* cpDNA encodes 112 unigenes, including 79 unique protein coding genes, 29 tRNA genes, four rRNA genes. Overall, 43.77%, 2.01%, and 6.80% of the genome sequence encode proteins, tRNAs, and rRNAs, respectively, whereas the remaining 48.02% are non-coding and filled with introns and intergenic spacers. The *O. rhizomatis* cp genome is AT-rich (60.99%), similar to other cp genomes (Raubeson et al. 2007; Gao et al. 2009; Yang et al. 2010), and the AT contents of protein-coding, tRNA, and rRNA are 60.57%, 47.47%, and 45.12%, respectively. Gene *matK* was located within the intron of *trnK-UUU*, and ycf68 was located within the intron of *trnI-GAU*. In *O. rhizomatis* cp genome, the pairs of genes *psbC-psbD*, *ndhC*-*ndhK* and *atpB-atpE* had 53-bp, 10-bp, and 4-bp overlapping regions, respectively. Eighteen genes contain introns, *ycf3* contains two introns, and the rest of the gene contains one intron; *rps12* is trans-spliced, one of its exons is in the LSC region (5′_end) and the two reside in the IR regions (3′_end) separated. A pair of gene *ndhH*, due to the 5′ part of *ndhH* which overlaps the IR/SSC junctions, was two unique genes. The four rRNA genes are all located in the IR. Twenty-one tRNA genes are located in the single-copy region. In the *O. rhizomatis* cp genome, the distance of *rps19* from the LSC/IR junction was 45 bp, while gene *ndhH* regions extended into the IR region in the junctions between IR and SSC.

The maximum likelihood (ML) phylogenomic analysis of the 14 complete cpDNA from the genus *Oryza* showed that *O. rhizomatis* is closely related to *O. officinalis*, they are CC genome types (Figure 1). The cpDNA sequences are increasingly used for resolving the deep phylogeny of plants because of their low rates of nucleotide substitutions and structural changes (Soltis et al. 2004; Jansen et al. 2007; Moore et al. 2010; Wu and Ge 2012). The new data will help to determine the phylogenetic placement of the genus *Oryza* and fill gaps in our understanding of *Oryzae* biology.

**Figure 1.**
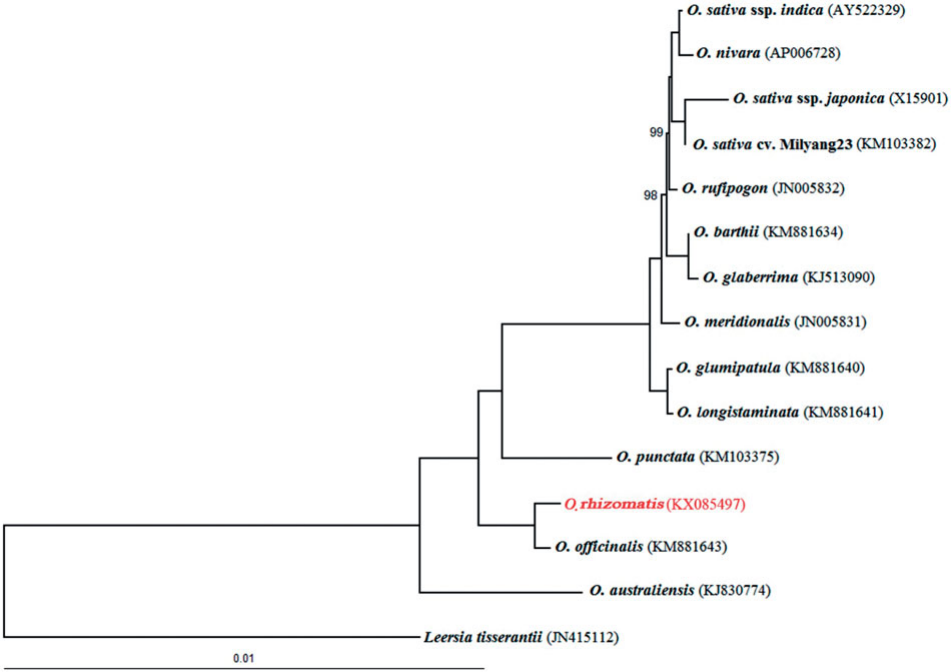
Maximum likelihood (ML) phylogeny of *Oryza* inferred from the whole-genome sequences of chloroplasts. Numbers near branches are bootstrap values of ML, the branches without numbers indicate 100% bootstrap supports. ML analyses were implemented in RAxML version 7.2.6 (Stamatakis 2006), GenBank accession numbers for sequences in brackets.
